# Measuring Athletic Mental Energy (AME): Instrument Development and Validation

**DOI:** 10.3389/fpsyg.2018.02363

**Published:** 2018-12-06

**Authors:** Frank J. H. Lu, Diane L. Gill, Cynthia M. C. Yang, Po-Fu Lee, Yi-Hsiang Chiu, Ya-Wen Hsu, Garry Kuan

**Affiliations:** ^1^Department of Physical Education, Chinese Culture University, Taipei, Taiwan; ^2^Department of Kinesiology, University of North Carolina, Greensboro, NC, United States; ^3^Graduate Institute of Physical Education, National Taiwan Sport University, Taoyuan, Taiwan; ^4^Department of Physical Education, Health and Recreation, National Chia-Yi University, Chia-Yi, Taiwan; ^5^Exercise and Sports Science, School of Health Sciences, Universiti Sains Malaysia, Kubang Kerian, Malaysia

**Keywords:** elite athletes, psychology of sports excellence, concentration, optimal state of mind, peak performance, psychological interventions

## Abstract

Although considerable research indicates that mental energy is an important factor in many domains, including athletic performance (Cook and Davis, 2006), athletic mental energy (AME) has never been conceptualized and measured. Therefore, the aim of this study was to conceptualize and develop a reliable and valid instrument to assess AME. In Study 1, a focus group interview established the initial framework of AME. Study 2 used a survey to collect athletes' experiences of AME and develop a scale draft titled “Athletic Mental Energy Scale (AMES).” In Study 3, we examined the psychometric properties and the underlying structure of AMES via item analysis, internal consistency, and exploratory factor analysis (EFA). In Study 4, we used confirmatory factor analysis (CFA) to examine AMES's factorial validity; and examined concurrent and discriminant validity by examining correlations with athletes' life stress, positive state of mind, and burnout. In study 5, we examined the measurement invariance of the 6-factor, 18-item AMES with Taiwanese and Malaysian samples. Study 6 examined the predictive validity by comparing AMES scores of successful and unsuccessful martial artists. Across these phases, results showed a 6-factor, 18-item AMES had adequate content validity, factorial structure, nomological validity, discriminant validity, predictive validity, measurement invariance, and reliability. We suggest future studies may use AMES to examine its relationships with athletes' cognition, affect, and performance. The application of AMES in sport psychology was also discussed.

## Introduction

Energy is a commonly-used word in research and general on the conversation. By its simplest definition, energy is “*the capacity for doing work* (Giancoli, 2009, p. 172).” Energy is important to human life because it allows us to satisfy our needs. Energy exists in diverse forms and comes from different sources, such as kinetic energy, chemical energy, solar energy, nuclear energy, and of particular interest in our research, mental energy.

Researchers in varied fields, including sport psychology, have studied mental energy. Especially, nutrition scientists are interested to examine what type of supplement enhances human mental energy. For example, in a study examining whether consumption of the sucromalt improves adults' perceptions of mental and physical energy, Dammann et al. (2013) adopted a double-blind, randomized, cross-over study to examine the effects of sucromalt on 44 healthy adults' mental energy. Results found sucromalt not only improves men's mental energy after 4–5 h of supplement but also a delay in mental fatigue. Similarly, to evaluate whether a chronic treatment of tryptophan-rich protein hydrolysate improves middle-aged women' emotional processing, mental energy levels, and reaction, Mohajeri et al. (2015) recruited 59 middle-aged women consumed tryptophan-rich protein hydrolysate by 0·5 g twice per day for 19 days. Results found tryptophan-rich protein hydrolysate improves emotional responses and cognitive function. Similar studies related to supplement of diverse nutrition on mental energy can be found in the studies of Ginkgo biloba (e.g., Kennedy et al., 2007; Snitz et al., 2009), Ginseng (e.g., Kennedy et al., 2004), Glucose (Reay et al., 2006), and Omega-3 (e.g., Johnson et al., 2008; Rogers et al., 2008).

Although nutrition scientists reported that supplements improved mental energy, they did not specifically define mental energy. Researchers have referred to mental energy as attention ability (e.g., Kennedy et al., 2007; Snitz et al., 2009; Mohajeri et al., 2015), reaction time (Mohajeri et al., 2015), memory (e.g., Kennedy et al., 2004, 2007), language (e.g., Snitz et al., 2009), visual processing speed (e.g., Reay et al., 2006; Kennedy et al., 2007), executive function (Snitz et al., 2009), or emotional experiences (e.g., Johnson et al., 2008; Quartiroli et al., 2018). Further, with no definitive definition of mental energy, researchers have used all sort of measures such as depression and anxiety scales (e.g., Rogers et al., 2008), memory tests (Kennedy et al., 2004), attention tests (Kennedy et al., 2004), mood scales (e.g., Johnson et al., 2008; Quartiroli et al., 2018), visual analog scales (Kennedy et al., 2004; Kuan et al., 2017), or self-developed questionnaires (e.g., Dammann et al., 2013; Kueh et al., 2018) to assess what they called “mental energy.”

Because of such diverse concepts and measures, psychology researchers have started to conceptualize mental energy. In a special issue of *Intelligence*, Lykken (2005) defined mental energy as…” *an individual's ability to continue long hours of thinking, concentrating attention, and blocking distractions to achieve a given task*.” Lykken (2005) proposed that great scholars such as Archimedes, Socrates, Galileo, Newton, and Einstein can create and produce so many astonishing works because they have strong mental energy. Further, Lykken (2005) contended that to achieve success we need an extraordinary abundance of mental energy.

Although Lykken (2005) proposed a preliminary definition of mental energy and described the important role of mental energy in human functioning, there is no solid framework for mental energy. To address this problem, the North American Branch of the International Life Science Institute (ILSI) initiated a workshop in a 2005 world conference to define and conceptualize mental energy. After discussion, they defined mental energy as “…*the intensity of subjective feeling about one's capacity to accomplish tasks of daily life-these feelings fluctuate from moment to moment* (O'Connor and Burrowes, 2006, p. 2).” The ILSI provided a preliminary model of mental energy that comprised 5 major components including motivation, cognition, quality of life, mood, and sleepiness (O'Connor and Burrowes, 2006, p. 2).

Built on this preliminary model of mental energy proposed by the ILSI, researchers proposed different measures of mental energy. For example, Lieberman (2006) proposed that researchers may use cognitive tests, mood questionnaires, electrophysiological indices, brain scanning, ambulatory monitoring, or peripheral markers (e.g., plasma, saliva, urine) to assess the cognitive dimension of mental energy. In contrast, O'Connor (2006b) suggested visual analog scales, Profile of Mood State (McNair et al., 1981), and the vitality scale of SF-36 (Ware, 2000) can assess the mood dimension of mental energy. Further, to assess the motivational aspect, Barbuto (2006) suggested using motivation scales and behavioral observations (e.g., goals accomplished, efforts and persistence) to provide the true picture of mental energy in motivation. As to the quality of life of the mental energy, researchers suggested SF-36 (Ware, 2000) or WHOQOL (Lucas-Carrasco, 2012) can be useful in assessing mental energy. Moreover, to assess the sleepiness dimension of mental energy, researchers suggested that Pittsburg Sleep Quality Index (PSQI) is a valid and reliable measuring tool (Nishiyama et al., 2014).

Sport psychology researchers have also been interested in mental energy and its relationship with the athletic performance, but mostly from anecdotal reports. For example, Nideffer (1985) described a psychological skill termed “*centering”* which can gather energy and lead to a state of being confident and focused. Nideffer (1985) used this skill to help a former men's javelin athlete set the world record. Nideffer (1985) proposed that when an athlete focuses on “ki” he/she will feel of state of energy, confidence, and stability. Similarly, Suinn (1986) contended that using “*Visual Motor Behavior Rehearsal*” can help athletes becoming focused, confident, and strong when facing competitions. Further, sport psychologists believe that athletes' performance is based on a pyramid structure of energy. At the bottom is physical energy, then emotional energy, mental energy, and at the top spiritual energy (Loehr, 2005). Among all types of energy, mental energy is associated with higher-order functioning (cognition, perception, abstract thinking, creativity, self-awareness/regulation).

Moreover, to achieve optimal performance state, sport psychologists teach athletes to identify their energy state by a “self-awareness checklist.” This self-awareness checklist includes a rating of energy state ranging from “1, had high energy” to “6, had low energy.” By doing so, athletes gain information about their optimal state and performance. Further, athletes can regulate their energy based on this self-monitoring records (Weinberg and Gould, 2015, p. 274). Recently, Sindik et al. (2015) attempted to develop a sport excellence scale. They used vigor (subscale of POMS) as a measure of mental energy, but they did not explain why they selected vigor as mental energy, or how they defined mental energy. Therefore, it can be concluded that sports researchers are concerned about mental energy. Therefore, it can be concluded that sports researchers are concerned about mental energy. Unfortunately, how mental energy should be defined, and how it should be measured are unknown.

The lack of a reliable and valid measure of mental energy in sports makes researchers difficult to advance their knowledge about the role of mental energy plays in sports settings. Especially, the fundamental questions such as what mental energy means to athletes? Is mental energy really predict athletic performance as Loehr (2005) hypothesized? If there is a mental energy in sports what should it be measured? Further, if mental energy is really important in influencing athletes' cognition, affect, and behavior as nutrition scientists indicated (Dammann et al., 2013; Mohajeri et al., 2015), what factors may increase mental energy for athletes, or what factors decrease mental energy for athletes? Further, if coaches or athletes intend to increase mental energy by what type of nutrition or training athletes can increase mental energy.

Given that there is a lack of mental energy measure in sports, the purpose of the present study was to develop a sport-specific mental energy scale. We adopted the guidelines suggested by the Standards for Educational Psychological Testing (American Educational Research Association, American Psychological, Association, and National Council on Measurement in Education, 2014) to conduct our research. In Study 1 we used a qualitative approach to provide a framework of AME; Study 2 adopted a survey approach to collect athletes' experiences of mental energy and develop a draft measure titled “Athletic Mental Energy Scale (AMES); study 3 examined the factor structure of AMES. Study 4 examined nomological and discriminant validity and Study 5 examined measurement invariance, and Study 6 examined the predictive validity of the AMES.

## Methods

### Study 1

#### Purpose

The purpose of Study 1 was to establish an operational definition and provide a framework of AME. To achieve this aim, a focus group interview was conducted.

#### Methods

##### Participants

Focus group participants were 11 experts in sport psychology (*n* = 4); physical education (*n* = 3); sports training and competition (*n* = 3); and sports sociology (*n* = 1) to provide a definition of AME, and their opinions/experiences of AME in order to generate a framework of AME.

##### Procedure

Prior to the interview, the researchers gained approval from a local institute ethical committee. Then, the first author contacted targeted experts through emails and phone calls and briefly informed them of the purpose of the research, confidentiality, and anonymity of their participation. After their agreement, we scheduled a group interview. Before the discussion, they signed informed consent. The interview questions focused on 3 major questions: (a) their general opinions about mental energy, and AME in particular; (b) their experiences as sport psychologists, physical education teachers, coaches, and sports professionals; (c) their definitions of AME. Each discussion was hosted by the first author, and the interview lasted for 2 h.

#### Results

The focus group interview generated 21,285 words and 201 meaningful themes. Inductive content analyses (Elo and Kyngas, 2007) revealed that experts described AME as an athlete's perception of his/her existing energy state, and it fluctuates moment to moment. Also, like the mental energy in general (i.e., Lykken, 2005) they mentioned that AME enables athletes to persist long hours of physical and mental efforts in athletic training and competition. They contended that the antecedents of AME including personal, social, and environmental factors. Also, they proposed that athletes' psychological states or life events may influence athletes' mental energy. The major results of the focus group are summarized in Table 1. Finally, the focused group interview concluded a tentative definition of AME as “*an athlete's perceived existing state of energy which is characterized by its intensity in motivation, confidence, concentration, and mood*.”

**Table 1:** Initial results of athletic mental energy (AME) by a focused group interview.

**Category**	**Descriptions**
General opinions of AME	As mental energy in other domains, AME enables athletes to perform well in athletic settings; AME is influenced by many personal and environmental factors such as life patterns, nutrition, sleep, interpersonal relationship, time management; AME can be gained from mental and physical training. As long as the athlete has good management in life they can regulate AME; AME is a key to athletic success; some mental training such as mindfulness, imagery, and goal setting is believed to be helpful in cultivating AME;
Experiences of AME	When low in AME will experience low in motivation; high in AME is also high in confidence; athletes will experience low anxiety when high in AME; becoming very concentrated when high in AME; feel cheerful and vigorous with sufficient AME; feel calm; tireless when high in AME; high in AME will be high in confidence to challenge tougher opponents, no worry, become tougher even in challenging situations, feel endless energy, full of passion in sports, feel invincible; smooth movement, flow, and clear goals;
Definitions of AME	AME is an athlete's perception about his/her existing energy state AME a state-like experience that is characterized by positive experiences such as high in vigor, concentration, confidence and motivation AME is an athlete's perceived energy state that enables athlete persists longer in training and competition AME is defined as an athlete' perception of his/her non-physical aspect of energy

#### Conclusion

The purpose of study 1 was to establish an operational definition of AME and an initial conceptual framework. The experts provided an operational definition, contents, antecedents, and consequences of AME. However, how athletes experience AME, and the components of AME are still unknown. Therefore, a field investigation of athletes was needed.

### Study 2

#### Purpose

The purpose of Study 2 was to collect athletes' experiences of mental energy and generate an item pool for the initial measure.

#### Methods

##### Participants

To generate an item pool of AME, we surveyed 242 college student-athletes (males = 137; females = 105; *M*_age_ = 20.62 years; SD = 1.87) about their experiences of having mental energy in athletic settings. At the time of the survey participants were engaged in competitive sports training and competitions. They participated either in individual sports such as golf, archery, track and field, swimming; or team sports such as basketball, volleyball, soccer, and baseball.

##### Measurements and Procedure

Prior to data collection, the researchers gained ethical approval from a local institute ethical committee. Then, the first author contacted targeted teams' coaches through emails and phone calls and briefly informed of the purpose of the research, the confidentiality, and anonymity of their participation. After agreements, we made an appointed date to collect data. A survey package included a demographic questionnaire and survey items- 4 open-ended questions asking (a) “when you perceive high energy during training and competition, what does it feel like?” (b) “when you perceive low energy during training and competition, what does it feel like?” (c) “what causes you to feel high in energy during training and competition,” and (d) “what causes you to feel low in energy during training and competition?” Participants provided responses to these questions based on their experiences in the blank space for each item.

#### Results

Initial analyses revealed 699 first-order themes. By using inductive content analysis (Elo and Kyngas, 2007) we generated 5 categories of AME, namely motivation, confidence, vigor, concentration, and calm Table 2 shows. We chose the 10 most frequently mentioned themes from each category to develop a 5-factor, 50-item athletic mental energy scale (AMES) draft. We used a 6-point Likert scale ranging from 1 (not at all) to −6 (completely so) to assess participants' responses on each item. The generic stem for the AMES was “At this moment (training or competition,) I feel that…” Sample items include “…endless energy comes from my body,” “…facing to up-coming competition I feel excited…,” and “…I have nothing to bother me in my mind.”

**Table 2:** Thematic analysis of athletes' experiences of mental energy.

**High Order themes**	**2nd order**	**Raw themes (frequencies)**
Athletic mental energy (AME)	Confidence	*Feel confidence (46) *I am sure I will win (13) *I am the best (9) *Feel confidence on all skills (24) *Feel invincible (3) *Feel flow in all movement (6) *Feel control over all sports movement (21) *I can surpass my opponents (7) *the more I compete the more I feel stronger (15) *I can accomplish all challenging tasks (9) *others (21)
	Motivation	*Clear goals to sports (34) *High motive to accomplish training (23) *Feel exciting on future competition (11) *Intensively engage in training (9) *Feel passionate to engage in sports (13) *Eager to compete (17) *Eager to show others *Expect much on future competition (15) *More involved in sports (8) *Expect to win (10) *Others (7)
	Vigor	*Feel energetic to do everything (32) *Feel endless energy comes from my body (21) *Feel tireless no matter how hard the training is (18) *Still feel energetic even after the competition (10) *Feel vigorous either in training or competition (9) *Feel stronger (7) *Feel full of combat will (15) *Feel powerful (12) *Feel explosive (6) *Feel adrenaline releasing (8) *Others (6)
	Concentration	*Becoming concentrated (33) *Feel free of distractions (21) *Deeply engage in sports (16) *Ignoring environmental distractions (7) *Clearly feel every moment (10) *Feel clear in mind (6) *Free from audience noises (9) *Automatic piloting of movements (10) *Free of outside voices (5) *Only hear my breathing in competition (6) *Others (8)
	Calm	*Feel no worry to compete (34) *No worry in mind (23) *Feel calm even compete with tougher opponents (19) *Feel calm for future competition (12) *Feel no pressure to compete (8) *Feel comfortable to compete (8) *Feel relaxed in competition (11) *Feel low anxiety in competition (4) *Free of fear in sports (7) *Feel relaxed in muscles (7) *Others (6)

Moreover, to examine the appropriateness of the 5-factor, 50 items AMES we invited 9 collegiate athletes to review all items for reading fluency, understandability, and true experiences. According to their review, several erroneous wordings and contradictions were replaced or modified. Therefore, we added 8 items in the vigor and motivation factors resulting in a 5-factor, 58-item AMES.

#### Conclusion

The purpose of study 2 was to collect athletes' experiences and construct an initial instrument for assessing AME. A survey and content check by college student-athletes produced a 5-factor, 58-item AMES. Generally, the five factors of AMES reflect the mental energy framework proposed by the ILSI. However, the 5-factor, 58-item AMES revealed several different categories and items. For example, ILSI proposed quality of life and sleepiness as two major categories of mental energy. However, in our 5-factor, 58-item AMES lacks such categories. Further, most items of the 5-factor, 58-item AMES reflect sport-specific experiences such as feeling, cognition, and behavior in training and competition. Whether the 5-factor, 58-item AMES possesses appropriate psychometric evidence such as internal consistency, factorial structure, criterion validity, and predictive validity is still unknown. Therefore, further field testing of the 5-factor, 58-item AMES was needed.

### Study 3

#### Purpose

The purpose of Study 3 was to explore the underlying factor structure of the 5-factor, 58-item AMES developed in Study 2.

##### Phase I

Pilot study.

Johnson and Brooks (2009) suggest that when constructing a new scale a pilot study can help researchers to estimate response rate and investigate the feasibility of a study. For this reason, before examining the underlying factor structure of the 5-factor, 58-item AMES we conducted a pilot study. The pilot study focused on two issues: (a) the appropriateness of the 5-factor, 58-item AMES in terms of language clarity and fluency; (b) basic psychometric properties of the 5-factor, 58-item AMES. We invited 15 college student-athletes to review the content of the 5-factor, 58-item AMES. Followed this procedure, we recruited 100 convenient sample of college student-athletes to examine the basic psychometric properties of the 5-factor, 58-item AMES. The 15 college student-athletes reported that the 5-factor, 58-item AMES is very straightforward, no ambiguous and abstract words, no double-barreled items, and no difficulties to read the contents. They can completely understand the contents of 5-factor, 58-item AMES. Further, a preliminary item discrimination analysis found that the mean of all items were between 2.49 and 4.43 (SD = 0.96 ~ 1.53), and skewness around −0.22 ~ 0.68 (kurtosis = −1.09 ~ 0.46). Also, there is a significant difference between high and low scores on all 58 items. Independent *t*-tests demonstrated that all 58 items exceeded the critical value of 4 (Kline, 1998). Therefore, the item discrimination is established. Thus, the pilot study suggested that 5-factor, 58-item AMES was suitable for further study.

##### Phase II

Formal study.

#### Methods

##### Steps to EFA

We adopted Williams et al. (2010) suggestions to conduct EFA including appropriate data, extraction method, criteria of factor extraction, selection of rotation method, and interpretation and labeling.

##### Participants

Participants in Study 3 were 243 college student-athletes (*M*_*age*_ = 20.30 ± 1.99; males = 150, females = 93) recruited from 13 universities in Taiwan. They had been participating in a variety of individual sports, such as golf, weight-lifting, archery, track and field, gymnastics, baseball, taekwondo, badminton; and team sports such as basketball, and volleyball with 6.72 ± 3.76 years of sports experiences. They trained an average of 3.43 h per day (*SD* = 1.31).

##### Measurements and Procedures

The procedures were the same as Study 2. Those interested in this study then signed informed consent forms and completed a survey package including a demographic questionnaire and 5-factor, 58-item AMES. The questionnaire took ~ 20 min to complete and was administered either before or after each team's training session. The measures included the following:

*Demographic Questionnaire*. The demographic questionnaire was designed to collect participants' age, gender, types of sports, and years of athletic experience.

*The 5-Factor, 58-Item AMES*. The contents, factors, rating scale, and sample questions as in Study 2.

##### Statistical Analyses

We screened all data by examining means, standard deviations, skewness, kurtosis, and outliers to make sure there were no abnormal data. Then, we performed an item analysis to examine whether there is a significant difference between high and low scores on all items. Following item analysis, we used Pearson product-moment correlation analysis to examine the relationships of all items. Followed we used Exploratory Factor Analysis (EFA) to examine the underlying structure of the 5-factor, 58-item AMES. Last we used Pearson product-moment correlation analysis to examine the relationships of factors extracted.

#### Results

Statistical Package for the Social Sciences version 18.0 (SPSS 18.0) was used for data screening and statistical analyses. Results indicated no outliers, the mean of all items were between 2.72 and 4.87 (SD = 0.01 ~ 1.31), and skewness around −0.00 ~ 0.60 (kurtosis = −0.02~−0.86) indicated that the raw data fit statistical assumptions. Item discrimination was calculated by comparing the items that were higher than one SD from the mean and items lower than one SD. Independent *t*-tests demonstrated that all 58 items exceeded the critical value of 4 (Kline, 1998). Further, Pearson product-moment correlation analysis found all items were correlated (*r* = 0.31 ~ 0.79) which exceed the minimum requirement of 0.30 for the subsequent EFA (Tabachnick and Fidell, 2007).

Further, before performing EFA, we checked Bartlett's test of sphericity (Bartlett's = 5020.65, *p* < 0.01), and Kaiser-Meyer-Olkin (KMO = 0.93), which showed that data were normally distributed and acceptable for factor analysis. We used the principal axes analysis method and oblique rotation to examine the underlying structure of the initial questionnaire because it is suggested that when the factors of a measure are conceptually correlated the oblique rotation is appropriate (Gorsuch, 1983, p. 203–204). With EFA parameters set at five factors and factor loadings exceeding 0.30 for solutions (Tabachnick and Fidell, 2007), 34 items were selected in the model. However, some items were either cross-factor loaded or selected into the wrong factors. We deleted these items and conducted a second round of EFA. Results revealed a 6-factor solution with 25 items accounting for 66.77% of the variance, as Table 3 indicates. Further, as Table 4 shows Pearson product-moment correlation analysis found all factors were correlated (*r* = 0.29 ~ 0.62) which are >*r* = 0.20 so all the items remained for further analysis (Child, 2006).

**Table 3:** AMES items and factor loadings from EFA.

**Item**	**COF**	**MOT**	**CON**	**TIR**	**COM**	**VIG**
9. I feel I can win all competitions in the future	0.75	–	–	–	–	–
14. I am tougher than my opponents	0.72	–	–	–	–	–
11. I am very confident to win the next competition	0.71	–	–	–	–	–
15. My sports movements and skills can be executed automatically	0.61	–	–	–	–	–
13. I can control all sports movements and skills	0.61	–	–	–	–	–
10. I can smoothly perform all sport skills	0.59	–	–	–	–	–
12. I am invincible, no one can beat me	0.59	–	–	–	–	–
21. I will try my best to get the best results in competitions	–	0.79	–	–	–	–
20. I want to show my best to others in sports	–	0.71	–	–	–	–
17. I am full of passion to attend my sports	–	0.67	–	–	–	–
16. I feel excited in future competitions	–	0.66	–	–	–	–
19. I can hardly wait to compete	–	0.64	–	–	–	–
24. I want to win all competitions in the future	–	0.61	–	–	–	–
29. There's nothing distracting me in competition	–	–	0.85	–	–	–
31. There's nothing distracting me in training	–	–	0.84	–	–	–
30. There's nothing I have to be care in competition/training	–	–	0.69	–	–	–
27. Even the training is over I still feel I have endless energy to use	–	–	–	0.82	–	–
26. Even the competition is over I still feel I have endless energy to use	–	–	–	0.75	–	–
28. No matter how long the training lasts I don't feel tired	–	–	–	0.63	–	–
34. Even facing to a tough opponent I don't feel anxious	–	–	–	–	0.80	–
33. Facing to coming competitions I don't feel anxious	–	–	–	–	0.76	–
32. When facing to my opponents I am calm	–	–	–	–	0.70	–
1. I feel spiritual to do everything in sports	–	–	–	–	–	0.81
5. I feel there is an endless energy coming from my body	–	–	–	–	–	0.67
8. Either in competition or training, I feel full of energy	–	–	–	–	–	0.55
Eigenvalues	3.88	3.49	2.55	2.46	2.28	2.03
% of Variance	15.50	13.97	10.20	9.84	9.12	8.13
Cumulative %	15.50	29.48	39.68	49.52	58.64	66.77
Cronbach's α	0.88	0.85	0.86	0.78	0.78	0.77

*COF, confidence; MOT, motivation; CON, concentration; TIR, tireless; COM, calm; VIG, vigor*.

**Table 4:** Correlation matrix and descriptive statistics for AMES factors.

	**Confidence**	**Motivation**	**Concentration**	**Tireless**	**Composed**	**Vigor**
1. Confidence	1.00	0.62^**^	0.51^**^	0.45^**^	0.58^**^	0.59^**^
2. Motivation		1.00	0.38^**^	0.42^**^	0.46^**^	0.60^**^
3. Concentration			1.00	0.51^**^	0.41^*^	0.42^**^
4. Tireless				1.00	0.29^**^	0.51^**^
5. Composed					1.00	0.38^**^
6. Vigor						1.00
Mean	24.65	25.99	10.03	8.94	10.89	11.32
SD	6.13	5.58	3.23	3.03	3.13	2.47
α	0.88	0.85	0.86	0.78	0.78	0.77

* p < 0.05,

** *p < 0.01*.

The six factors were: (a) vigor (VIG, Cronbach's α = 0.77) with items 1, 5, and 8; (b) confidence (COF, Cronbach's α = 0.88) with items 9, 14, 11, 15, 13, 10, and 12; (c) motivation (MOT, Cronbach's α = 0.85) with items 21, 20, 17, 16,19, and 24; (d) tireless (TIR, Cronbach's α = 0.78) with items 27, 26, and 28; (e) concentration (CON, Cronbach's α = 0.86) with items 29, 31, and 30; and (f) calm (CLM, Cronbach's α = 0.78) with items of 34, 33, and 32. The tireless factor was a new factor that was not found in Study 2.

#### Conclusion

The purpose of Study 3 was to examine the underlying factor structure of the 5-factor, 58-item AMES. A pilot study found participants can easily understand the contents of the 5-factor, 58-item AMES. Therefore, the content validity of the 5-factor, 58-item AMES was established. Further, a preliminary examination found that the 5-factor, 58-item AMES had significant discriminant indices on all items which indicated that the 58-item AMES can differentiate high vs. low scores. The correlation matrix also found all items correlation coefficients exceed 0.30 suggested that all items are acceptable for subsequent EFA. Further, we adopted (Worthington and Whittaker, 2006, p. 822–823) to remove those items which failed to contribute meaningfully of the scale such as low factor loadings, cross-loading, or inappropriate to represent the conceptual model of AME. Finally, the two rounds of EFA showed that the 6-factor, 25-item AMES had the best solution for the factor structure and reliability. Study 3 achieved the initial goal of gaining a basic tool for assessing AME in sports. However, there is a lack of other psychometric evidence such as the consistency of the factorial structure and criterion validity of the 6-factor, 25-item AMES. Therefore, further study is needed.

### Study 4

#### Purpose

The purpose of Study 4 was to confirm the factor structure of the 6-factor, 25-item AMES by Confirmatory Factor Analysis (CFA) and examine nomological validity by examining the relationships among AMES subscales, positive state of mind, college student-athletes' life stress, and burnout. Also, we examined the discriminant validity by comparing Square root of AVEs and coefficients of all 6 factors of AMES (Netemeyer et al., 1990).

#### Methods

##### Participants

Participants in Study 4 were 312 college student-athletes (*M*_*age*_ = 19.87, SD = 1.54, male = 208; female = 104) recruited from 14 universities in Taiwan.

##### Measurements and Procedures

The procedures were the same as Study 2 and 3. Those interested in this study then signed informed consent forms and completed a survey package including a demographic questionnaire, 6-factor, 25-item AMES, positive state of mind, college student-athletes' life stress scale, and burnout as follow:

*Athlete Burnout (ABQ).* ABQ (Raedeke and Smith, 2001) is a self-reported inventory that assesses athletes' burnout experiences. Raedeke and Smith (2001) reported that ABQ has three subscales including (a) the reduced sense of athletic accomplishment, (b) perceived emotional and physical exhaustion, and, (c) the devaluation of sports participation. To evaluate athletic burnout experiences participants were asked to answer the questions of ABQ in a 6-point Likert scale that ranged from 1 (*never*) to 6 (*always*). In the present study, the Cronbach's α for the three subscales ranged from 0.73 to 0.87 and the reliability for all items was 0.91.

*College Student-Athletes' Life Stress Scale (CSALSS).* The 24-item CSALSS (Lu et al., 2012) was used to assess situations that athletes encountered in their daily life and sports and considered as major stressors in their lives. There are eight factors in the 24-item CSALSS including: (a) sports injury, (b) performance demand, (c) coach relationships, (d) training adaptation, (e) interpersonal relationships, (f) romantic relationships, (g) family relationships, and (h) academic requirements. Lu and colleagues (Lu et al., 2012) reported that CSALSS can be categorized into two major components—general life stress (by adding factor e, f, g, h) and sport-specific stress (by adding factor a, b, c, d). Participants indicated the frequency of the event on a 6-point Likert scale ranging from 1 (*Never*) to 6 (*Always*). Cronbach's α of CSALSS in this study for general life stress was 0.84 and sport-specific stress was 0.86, indicating that the scale was reliable.

*Athletic Positive State of Mind Scale (APSMS).* APSMS (Chang and Lu, 2002) was adapted from Horowitz et al. (1988) Positive States of Mind (PSOM). Chang and Lu (2002) adapted the original six items of PSOM by replacing the statement of the item's stem into sports-specific questions and providing preliminary reliability and validity through item analysis, EFA, and CFA. These six major elements include attentional focus, productivity, maintaining responsibility, restful repose, and sensual pleasure. Data in the present study supported the single factor structure and the Cronbach's α was 0.87.

##### Statistical Analyses

We screened all data as described in study 3. Then, we used AMOS version 22 to check multivariate normality and perform a CFA analysis by the following criteria: (1) the χ^2^/DF ratio (between 1 and 3); (2) the root mean square error of approximation (RMSEA, < 0.08); (3) the standardized root mean square residual (SRMR, < 0.05); (4) the Goodness of Fit Index (GFI, >0.90); (5) the Comparative Fit Index (CFI, >0.90) (Hu and Bentler, 1999; McDonald and Ho, 2002). The composite reliability (CR > 0.70) (Hair et al., 2014) and average variance extracted (AVE > 0.50) (Hair et al., 1998) were calculated to examine the fit of internal structure.

#### Results

**Figure 1:**
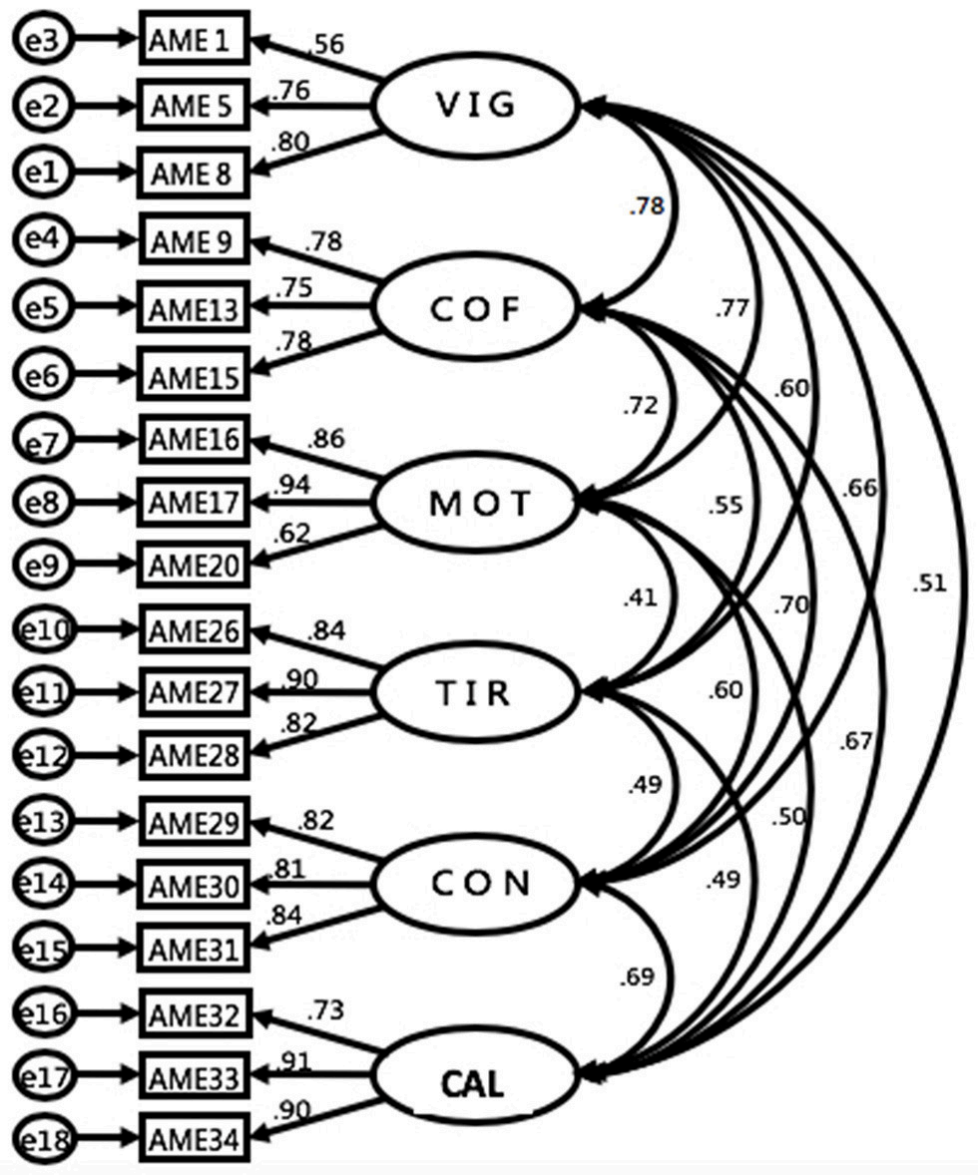
The 6-factors measurement model of the Athletic Mental Energy.

The univariate normality examination found no outliers and the mean of all items were between 5.08 and 1.65 (SD = 1.90 ~ 0.94); the skewness was around 1.55 ~ 0.06 (kurtosis = 2.24~−0.03). Further, multivariate normality examination found Mardia's normalized estimate was < 3 which indicate that the assumption of multivariate normality is met (Bentler and Wu, 2002). Further, the CFA analysis trimmed 7 items and found that the measurement model of the 6-factor, 18-item AMES was satisfactory according to related indices (RMSEA = 0.06, SRMR = 0.05, χ^2^/DF = 269.15, CFI = 0.96, GFI = 0.92, TLI = 0.946). The factor loadings for the 18 items ranged from 0.62 to 0.84 (Figure 1). The composite reliability (Fornell and Larcker, 1981) for each subscale was calculated: vigor (0.75), confidence (0.82), motivation (0.86), tiredness (0.89), concentration (0.87), and calm (0.89), indicating that each was above the.70 standard (Hair et al., 2014). The average variance extracted was also calculated: vigor (0.51), confidence (0.60), motivation (0.67), tiredness (0.73), concentration (0.68), and calm (0.72) which exceed cut-off value of 0.50 (Hair et al., 2014). Further, as Table 5 showed AME positively correlated with the positive state of mind but negatively correlated with athlete burnout and life stress which evidenced the nomological validity. Further, as Table 6 shows the Square root of AVEs is greater than correlation co-efficient among 6 factors of AMES. Thus, this indicated that 6-factor, 18-item AMES shows discriminant validity.

**Table 5:** The bivariate correlations of AME, APSMS, ABQ, and CSALSS.

	**1**	**2**	**3**	**4**	**5**	**6**	**7**	**8**	**9**	**10**	**11**	**12**	**13**	**14**
1.AME	**0.93**													
2.APSMS	0.58^*^	**0.87**												
3.ABQ	−0.32^*^	−0.27^*^	**0.91**											
4.CSALSS-sport	−0.30^*^	−0.23^*^	0.48^*^	**0.86**										
5. CSALSS-life	−0.24^*^	−0.25^*^	0.51^*^	0.69^*^	**0.84**									
6.AME-Vi	0.72^*^	0.53^*^	−0.29^*^	−0.31^*^	−0.30^*^	**0.73**								
7.AME-Cof	0.84^*^	0.53^*^	−0.23^*^	−0.24^*^	−0.17^*^	0.56^*^	**0.80**							
8.AME-Mot	0.77^*^	0.41^*^	−0.27^*^	−0.14^*^	−0.12	0.55^*^	0.64^*^	**0.83**						
9.AME-Tir	0.70^*^	0.35^*^	−0.31^*^	−0.31^*^	−0.21^*^	0.45^*^	0.49^*^	0.38^*^	**0.87**					
10.AME-Con	0.81^*^	0.44^*^	−0.22^*^	−0.25^*^	−0.18^*^	0.52^*^	0.60^*^	0.54^*^	0.41^*^	**0.88**				
11.AME-Cal	0.82^*^	0.48^*^	−0.18^*^	−0.15^*^	−0.16^*^	0.44^*^	0.63^*^	0.54^*^	0.46^*^	0.67^*^	**0.90**			
12.ABQ-RA	−0.35^*^	−0.36^*^	0.80^*^	0.39^*^	0.48^*^	−0.32^*^	−0.35^*^	−0.33^*^	−0.21^*^	−0.25^*^	−0.22^*^	**0.73**		
13.ABQ-E	−0.22^*^	−0.16^*^	0.89^*^	0.45^*^	0.42^*^	−0.18^*^	−0.13^*^	−0.12	−0.36^*^	−0.13^*^	−0.11	0.54^*^	**0.87**	
14.ABQ-D	−0.27^*^	−0.21^*^	0.89^*^	0.40^*^	0.44^*^	−0.28^*^	−0.17^*^	−0.29^*^	−0.21^*^	−0.21^*^	−0.16^*^	0.63^*^	0.68^*^	**0.86**
Mean	73.84	29.15	33.97	28.60	25.28	13.20	12.21	14.03	10.54	11.91	11.90	10.17	14.13	9.67
SD	13.52	5.45	11.45	9.33	10.26	2.20	2.74	2.72	3.37	3.11	3.26	3.53	5.28	4.43

* *p < 0.05*.

*Cronbach alphas are presented on the diagonal as bold font*.

*AME, total score of athletic mental energy; APSMS, athletic positive state of mind scale; ABQ, total score of athlete burnout questionnaire; CSALSS-sport, sport-specific life stress; CSALSS-life, general-life stress; AME-Vi, vigor; AME-Cof, confidence; AME-Mot, motivation; AME-Tir, tireless; AME-Con, concentration; AME-Cal, calm; ABQ-RA, reduced sense of athletic accomplishment; ABQ-E, emotional and physical exhaustion; ABQ-D, devaluation of sports participation*.

**Table 6:** Discriminant validity of the 6-factor, 18-item AMES.

	**Vigor**	**Confidence**	**Motivation**	**Tireless**	**Concentration**	**Composed**
Vigor	**0.713**					
Confidence	0.781	**0.773**				
Motivation	0.770	0.715	**0.817**			
Tireless	0.600	0.554	0.410	**0.853**		
Concentration	0.661	0.696	0.604	0.494	**0.826**	
Composed	0.512	0.671	0.505	0.493	0.692	**0.849**

*Square root of AVES in bold are on diagonals. Off diagonals are Pearson Correlations of all 6 constructs of AMES*.

#### Conclusion

The purpose of study 4 was to confirm the factor structure of the 6-factor, 25-item AMES from Study 3 and examine concurrent and discriminant validity by examining the relationships among AMES subscales, positive state of mind, college student-athletes' life stress scale, and burnout. By sampling 312 university athletes and administering the 6-factor, 25-item AMES, CFA analysis found that the measurement model of the 6-factor, 18-item AMES was satisfactory according to related indices. Further, bivariate correlations found AME positively correlated with the positive state of mind but negatively correlated with athlete burnout and life stress which provided nomological validity. Further, Square root of AVEs is greater than correlation coefficients among 6 factors of AMES. Thus, this indicated that 6-factor, 18-item AMES shows discriminant validity.

Through these stages, it seemed that the 6-factor, 18-item AMES shows adequate factorial structure, nomological validity, discriminant validity, and reliability. However, the construct validity used test scores and criterion scores at the same time. It is suggested that examining the test scores and criterion scores at a later time would provide evidence of predictive validity (American Educational Research Association, American Psychological, Association, and National Council on Measurement in Education, 2014, p. 28). Also, it is suggested that a well-developed measurement should present measurement invariance so it can indicate that the same construct is being measured across some specified groups (Widaman et al., 2010). Hence, the predictive validity of 6-factor, 18-item AMES, and its measurement invariance are needed.

### Study 5

#### Purpose

The purpose of study 5 was to examine measurement invariance of the 6-factor, 18-item AMES across Malaysian and Taiwanese samples.

#### Methods

##### Participants and Procedures

Participants in Study 5 were from new data of Malaysian (*n* = 156, *M*_*age*_ = 19.61, SD = 2.20, males = 88; females = 68) and Taiwanese samples (*n* = 223, *M*_*age*_ = 20.21 ± 1.19; males = 147, females = 76).

##### Measurements

The measures included the demographic questionnaire and the 6-factor, 18-item AMES.

##### Statistical Analyses

We used AMOS version 22 to perform a measurement invariance. We adopted earlier suggestion (Barbosa-Leiker et al., 2011) by following procedures: (a) once the confirmatory factor models for each group established that the overall model was acceptable, a series of analyses to examine measurement invariance were performed sequentially between comparison and nested model; (b) each model was added equality constraints and was tested against the less-constrained model including configural invariance, metric invariance, factor variance/covariance invariance and error variance invariance (Horn and McArdle, 1992).

For tests of invariance, χ^2^ difference tests are typically used to compare nested models. However, the χ^2^ difference test may also be influenced by sample size (Chen et al., 2005); thus, a change in the comparative fit index (CFI) between comparison and nested models of greater than or equal to 0.010. In addition, we examined the change in root mean square error of approximation (RMSEA) 0.015 or a change in standardized root mean square residual (SRMR) 0.030 (for loading invariance) and 0.010 (for intercept invariance) is recommended as an appropriate criterion indicating a decrement in fit between models (Cheung and Rensvold, 2002; Chen et al., 2005; Chen, 2007). Additionally, a χ^2^ difference test for a small difference between models (rather than 0) was also conducted (MacCallum et al., 2006).

#### Results

Table 7 shows the Malaysian and Taiwanese 6-factor, 18-item AMES model of the measurement invariance, M1 was configuration invariance model, M2 metric invariance, M3 variation\covariance invariance, M4 error variance invariance is shown to have acceptable adaptation indicators. ΔCFI indicated that 6-factor 18-item model of Malaysian and Taiwanese in M1, M2, M3 measurement invariance model display equivalent (ΔCFI ≤ 0.01), however, M4 shows the residuals are not equal (ΔCFI > 0.01). We will discuss this later in the discussion.

**Table 7:** Measurement Invariance Models of the AMES between Malaysian and Taiwanese.

		***df***	**χ^2^**	**CFI**	**RMSEA**	**SRMR**	**ΔCFI**
Malaysian v.s. Taiwanese	M1 (configural )	240	380.65^*^	0.959	0.038	0.0469	
	M2 (metric)	252	406.11^*^	0.955	0.039	0.0574	−0.004
	M3 (variance\covariance)	273	462.44^*^	0.945	0.042	0.0853	−0.010
	M4 (residual)	291	729.20^*^	0.872	0.056	0.0960	−0.073

* *p < 0.001*.

#### Conclusion

The purpose of Study 5 was to examine the measurement invariance of the 6-factor, 18-item AMES. We found configuration invariance, metric invariance, and variance/covariance invariance that were all equivalent except error variance invariance. Therefore, it means that the same level of measurement error for each item between Malaysian and Taiwanese is not the same. However, Lee (2006) suggested that most research that using CFA focus on the equivalence of the factor loadings and factorial covariance. If these indicators meet criteria they can assure that means measurement invariance across observed groups is held, while residual restrain model may be too critical to be reached. Tabachnick and Fidell (2007) also suggest that when factor loadings and factorial covariance are equivalent across group it is indicated that measurement invariance holds true.

### Study 6

#### Purpose

The purpose of study 6 was to examine the predictive validity of the 6-factor, 18-item AMES.

#### Methods

##### Participants and Procedures

Another new sample (*n* = 78) was recruited from the Malaysian Chinese university male martial artists (*M*_age_ = 19.28 yrs, *SD* = 2.01) from the 2018 Malaysian Intervarsity Wushu Championships. The competitions were recognized by the Wushu Federation of Malaysia, and the events include *Chang Quan, Nan Quan, Tai Ji Quan, Dao Shu, Nan Dao, Qiang Shu, Jian Shu, Tai Ji Jian, Gun Shu, Nan Gun, San Shou*, and *Chuan Tong Tai Ji Quan*. The sample size was determined based on the statistical test of logistic regression, by using G^*^Power 3.1.7. With an expected off ratio of 2.5, alpha of 0.05, the power of 0.80, the estimated sample size was 70. After adding the estimated drop-out rate of 15%, we required a total sample size of 81, however, in this study, due to the competition environment, only 78 participants volunteered to participate in this study.

The participants completed the demographic questionnaire and six-factors, 18-item AMES a day before the championship. Then, after completion of the championship, we collected their competition records of medals. In this study, winning a medal in the competition was considered as successful performance outcome. The study received approval from the Universiti Sains Malaysia (USM) Human Research Ethics Committee (USM/JEPeM/812149) and was conducted in accordance with the guidelines of the International Declaration of Helsinki. The general procedures (i.e., ethical approval, contacted coaches, briefing the research, completed consent form and questionnaire package) for the testing were similar to those of previous sections.

##### Measurements

The measures included the demographic questionnaire and the six-factors, 18-item AMES. In this sample, the Cronbach's α for each factor was vigor (0.73), confidence (0.62), motivation (0.67), tiredness (0.86), concentration (0.88), calm (0.86), and for total AMES (0.95).

##### Statistical Analyses

We used SPSS 24.0 to analyze the data. Specifically, we used logistic regression to examine whether AME predicted winning medals (Harrell, 2001). Firstly, simple logistic regression was used to examine the association between individual independent variables with the dependent variables (Medal and non-Medal). Then multiple logistic regression was used to determine the significant independent variables in the binary logistic regression model. The crude odd ratio and adjusted crude odd ratio and its 95% confidence intervals (CI), Wald statistic and *p*-value were reported in the results.

#### Results

Table 8 shows the simple and multiple logistic regression results of the associations between total score and subscale of AMES and the outcome of medal and non-medal recipients. The results showed that AMES was associated with the outcome of medal and non-medal. Specifically, an increase in 1 unit score of AMES was associated with 1.14 times the odds to win a medal when adjusted for AMES. Also, the results of multiple logistic regression with the subscales of AMES showed that confidence, motivation, tireless, and calm were associated with the medal and non-medal outcome. A person with an increase in 1 unit score in confidence has 2.06 times the odds of getting a medal. An increase in 1 unit score in motivation had 1.55 times odds of getting a medal. An increase in 1 unit score in tireless had 47% lower odds to get a medal. And, an increase in 1 unit score in calm had a 1.79 times odds of winning a medal.

**Table 8:** Factors associated with total score and subscales of AMES with medal and non-medal.

**Variable**	**Simple logistic regression**				**Multiple logistic regression**			
	**Regression coefficient (b)**	**Crude Odd Ratio (95% CI)**	**Wald stat**.	***p-*value**	**Regression coefficient (b)**	**Crude odd ratio (95% CI)**	**Wald stat**.	***p-*value**
**AMES**								
Confidence	0.66	1.94 (1.38, 2.74)	14.41	< 0.001	0.72	2.06 (1.20, 3.55)	6.82	0.009
Motivation	0.45	1.57 (1.19, 2.07)	9.96	0.002	0.44	1.55 (1.01, 2.36)	4.09	0.043
Tireless	0.09	1.10 (0.93,1.30)	1.17	0.279	−0.63	0.53 (0.32,0.88)	5.86	0.016
Composed	0.48	1.62 (1.24, 2.12)	12.45	< 0.001	0.58	1.79 (1.18,2.72)	7.54	0.006
Total AMES	0.12	1.12 (1.05, 1.20)	11.17	0.001	0.13	1.14 (1.06, 1.23)	11.55	0.001

#### Conclusion

The purpose of study 6 was to examine the predictive validity of the six-factors, 18-items AMES with Malaysian University Chinese martial artists. Logistic regression results showed total AMES and four factors of AME-confidence, motivation, tireless, and calm predicted medal winning. Thus, the predictive validity of the 6-factor, 18-items AMES was supported.

## Discussion

By adopting the ILSI framework of mental energy and following the guidelines suggested by the Standards for Educational Psychological Testing (American Educational Research Association, American Psychological, Association, and National Council on Measurement in Education, 2014) this research produced a sport-specific mental energy scale entitled “Athletic Mental Energy Scale (AMES). Specifically, across five studies, we found a 6-factor, 18-item AMES had appropriate content validity, factor structure, convergent validity, discriminant validity, predictive validity, and reliability.

Generally, the 6-factor, 18-item AMES reflects the basic framework of the ILSI but includes several unique components pertaining to sports. Our study found that AME comprises 6 factors: vigor, motivation, confidence, tireless, concentration, and calm. The factor of motivation is similar to ILSI framework, but our study found three factors (i.e., vigor, tireless, and calm) to replace mood of the ILSI framework. Further, our study found another two factors—self-confidence and concentration to replace the ILSI framework of cognition. Moreover, our study did not include sleep and quality of life into the framework but we found a unique factor “calm” that is not included in the ILSI framework. Thus, our study is in line with the ILSI framework but creates a unique model of mental energy specific to the sport.

## Strengths of the Study

The strength of this study was in adopting existing ILSI framework for mental energy and taking an empirical approach to produce a reliable and valid sport-specific mental energy scale. Our study followed The Standards for Educational Psychological Testing suggestions for developing a measure of an individuals' knowledge, skills, abilities, interests, attitudes, or other characteristics (American Educational Research Association, American Psychological, Association, and National Council on Measurement in Education, 2014, p. 75–93). We designed and followed a specific plan through six studies from the qualitative exploration of AME to psychometric testing of measurement validity and reliability. With this approach, we believe we have improved existing mental energy measures such as Kennedy et al. (2004); Rogers et al. (2008); Johnson et al. (2009); Dammann et al. (2013) in nutrition science, and Sindik et al. (2015) in sports. The AMES provides a reliable and valid instrument for research on AME.

## Theoretical Contributions/Implications

The AMES is the first measure of mental energy in sports and psychology with sufficient reliability and validity. By this tool, researchers can not only help researchers to examine what factors lead to AME but also examine how AME influence athletes' cognition, emotion, and performance. Also, our preliminary work on AME has several theoretical implications for researchers. First, the definition of AME reflects Lykken (2005) and ILSI (O'Connor, 2006a) works. Lykken (2005) defined mental energy as “*an individual's ability to continue long hours of thinking, concentrating attention, and blocking distractions to achieve a given task*,” and the ILSI defined mental energy as “…*the intensity of subjective feeling about one's capacity to accomplish tasks of daily life-these feelings fluctuate from moment to moment*.” Our study defined AME as “*an athlete's perceived existing state of energy, which is characterized by its intensity in motivation, confidence, concentration, and mood*.” Our definition not only extends the ILSI's conceptualization of the mental energy but also denotes AME as a multi-dimensional construct pertaining to sporting experiences.

The affective components (i.e., vigor, tireless, and calm) of AME echo peak performance research in sport psychology. For example, Morgan (1979, 1980) used profile of mood state (POMS) to assess U.S. Olympic rowing, swimming, and wrestling candidates and found successful athletes scored high on vigor but low on anxiety, fatigue, depression, anger, and confusion compared to unsuccessful athletes. Similarly, the calm factor of AME also supports Ravizz's (1977) findings that athletes reported they had no fear of loss, and feeling physically mentally relaxed when they performed their best. Further, Loehr (1984) found athletes who performed extraordinarily well-experienced seeming controlled by competition, full of energy but in an easy manner. Recently, Fletcher and Sarkar (2012) investigated resilience in Olympic gold medalists found Olympic champions possess several unique characteristics such as open to new experiences, emotional stable, and optimistic. The calm and relaxation experiences are also frequently found in flow research (e.g., Csikzentmihalyi, 1990; Jackson, 2011). When individuals experience flow they reported sensations of relaxation, calm, and effortless. Thus, the affective components of AME seemed linking to peak performance experiences and flow.

The cognitive components (i.e., confidence and concentration) of AME support athletes' experiences in peak performance too. Self-confidence represents athletes' beliefs about whether they can accomplish the task (Vealey and Chase, 2008). Literature has demonstrated a consistent relationship between high levels of confidence and successful sporting performance (Feltz et al., 2008). Past research on peak performance found when athletes were playing their best they are highly confident with no fear of failure (e.g., Ravizz, 1977; Garfield and Bennett, 1984; Loehr, 1984). Also, when performing their best athletes reported that they were totally concentrated, with a narrow focus of attention on the present, immersed in the activity, and completely in control while acting. In a recent study on the optimal psychological state for peak performance, Anderson et al. (2014) found athletes reported that automatic execution of performance, present moment thinking, focused, and clear mind were the most frequently identified experiences. Thus, the cognitive components of AME coincide with the findings in peak performance research.

The component of motivation in AME is relevant to sport psychology. It is estimated that one-third of all studies in sport psychology deal with motivation in one form or another (Roberts, 2012). Thus, it is not surprising that ILSI and our study identified motivation as one of the core factors of AME. The motivation component in AME mostly refers to athletes' expectation and goals in sports (e.g., I want to show my best to others in sports). Thus, it is suggested that AME would influence athletes' investment in sports training and competition. Those low in AME would be expected to be low in sports engagement (e.g., attendance, persistence, efforts, intensity, and choice of a challenge). Therefore, it is likely that any motivational intervention (e.g., Joesaar et al., 2012) would influence athletes' AME. However, further research is needed to confirm these inferences.

The nomological validity findings provide information for researchers and practitioners. Specifically, we found AME positively correlated with the positive state of mind. According to Horowitz et al. (1988) positive state of mind represents one's psychological state free of worry, focused, caring, and pay attention to his/her work at hand. Hence, it is implied that athletes high in AME would be high in the positive state of mind. In contrast, we found AME negatively correlated with athletic burnout and life stress. Past research indicated that athletes high in positive psychological attributes—such as self-confidence (e.g., Federici and Skaalvik, 2012), counter stress (Chyi et al., 2017), optimism (e.g., Gustafsson and Skoog, 2012), hope (e.g., Gustafsson et al., 2013) had lower levels of burnout. Hence, the present study not only supports past research on the relationship between athletes' positive attributes and burnout but also suggests AME has an influence on burnout.

The predictive validity findings have several theoretical implications. To the best of our knowledge, this is the first study examining how AME predicts athletic performance. For a long time, sport psychology researchers have been interested in psychological profiles that predict athletic performance. Many hypothetical models, for example Morgan's (1979, 1980) iceberg profile, Hanin (2000) Individual Zone of Optimal Functioning (IZOF), psychological skills (Mahoney et al., 1987), performance strategies (Thomas et al., 1999), mental skills (Durand-Bush et al., 2001), mental toughness (Jones et al., 2007), and resilience (Fletcher and Sarkar, 2012) have been proposed. The present study adds to this literature and suggests that AME predicts athletic performance. However, only four factors -confidence, motivation, tireless, and calm were the significant predictors of martial artists' success. The factors of confidence, motivation, tireless, and calm support past research on peak performance (e.g., Ravizz, 1977; Garfield and Bennett, 1984; Loehr, 1984), but the role of other two factors—vigor and concentration need to be examined in the future.

## Limitations and Future Suggestions

### Limitation

Our study had several limitations. First, although we used a qualitative approach to establish an initial framework of AME, our study only offers a preliminary framework and future study is needed. We suggest future adopting the same approach of the present study and exploring the potential model of AME. Second, our participants were all recruited from Taiwan and Malaysia, and the participants are mostly Asians. Therefore, AME needs to be examined in different continents and cultures. Further, the samples were all college student-athletes, and our results can't be generalized to professional athletes or younger athletes. Furthermore, since the AMES is a state-like measure it is very sensitive to situational and contextual factors (Rule and Traver, 1983; Jacobs et al, 1988) we did not examine its test-retest reliability. We suggest future study may examine the fluctuation of AME to examine its relationship with environmental and personal factors. Moreover, on Study 3 we used 243 college student-athletes as our participants to perform EFA. Though it is acceptable in related literature. For example, Hair et al. (1998) suggested that the sample size should be 100 or greater. However, Comrey and Lee (1992) suggested that sample size as 100 is poor, 200 is fair, 300 as good, 500 as very good, and 1,000 or more as excellent. We suggest future study should recruit more participants when performing EFA.

### Future Research Suggestions

We suggest that future research may validate the 6-factor, 18-item AMES in different cultures. Another line of future research might examine the antecedents and consequences of mental energy. For example, supplements of sucromalt (e.g., Dammann et al., 2013) and tryptophan-rich protein hydrolysate (Mohajeri et al., 2015) were found to increase mental energy. Whether these supplements also increase AME needs further examination. In addition, we suggest future study may examine what psychological skill training increases AME. In particular, recent studies found mindfulness training increases athletes' concentration and confidence (e.g., Kee et al., 2012; Jouper and Gustafsson, 2013). Therefore, whether mindfulness training also enhances AME needs further examination. Further, research suggested that insufficient sleep influences athletes' memory, cognition, and performance (Halson, 2014). Thus, whether insufficient sleep also influences AME can be another direction for researchers. Moreover, we suggest future study may examine the associations of AMES and other sports behavior such as coach-athlete relationship (Jowett and Poczwardowski, 2007), sport confidence (Beaumont et al., 2015), flow and optimal performance (Jackson, 2011), attention and frontal midline theta activity (Kao et al., 2014), and sport motivation (Pelletier et al., 2013).

Further, through 6 studies we have established the preliminary psychometric properties of the 6-factor, 18-item AMES by examining the content validity, factor validity factorial structure, nomological validity, discriminant validity, predictive validity, measurement invariance, and reliability, there are remaining many spaces for future researchers to examine the underlying psychometric properties of AMES. For example, future research may compare the group differences between high and low-performance athletes to establish predictive validity. Also, researchers may conduct a laboratory or filed experiment to examine how AME influence athletic performance to examine construct validity of the 6-factor, 18-item AMES. Moreover, researchers may adopt a cross-cultural approach to examine the factorial validity, construct validity, and predictive validity in different cultures.

### Applications

In terms of application, we suggest coaches or sport psychology consultants may use this 6-factor, 18-item AMES to monitor athletes' training loadings and AME. Research suggests that excessive training loads increase athletes' anger, anxiety, depression, and fatigue (Raglin et al., 1995, 1996). Therefore, coaches or sport psychology consultants might use AMES to monitor athletes' training status to help athletes with appropriate training. In a similar way, coaches or sports scientists may use AMES to monitor athletes' training loadings in pre-season or in-season (Jeong et al., 2011). Specifically, the AMES can help coaches or sports scientists understand whether changes in training loadings influence athletes' mental energy. Further, it is recommended that sport psychology consultants may use AMES to assess athletes' mental strength and weakness before conducting a psychological skills training (PST) (Weinberg and Williams, 2013, p. 338–339).

## Conclusion

To obtain a reliable and valid measure of the AME, we conducted five studies and developed a 6-factor, 18-item AMES. We think this is just a starting point of the AME research in sports settings. We hope our study inspires more research on this issue, not only for the pursuit of knowledge but also for the exploration of psychological factors underlying sport excellence.

## Ethics Statement

The research has been approved by the Antai-Tian-Sheng Memorial Hospital Institutional Review Board (TSMH IRB No 16-025-B), and Universiti Sains Malaysia (USM) Human Research Ethics Committee (USM/JEPeM/18050226).

## Author Contributions

All authors listed have made a substantial, direct and intellectual contribution to the work, and approved it for publication.

### Conflict of Interest Statement

The authors declare that the research was conducted in the absence of any commercial or financial relationships that could be construed as a potential conflict of interest.
